# Zimbabwean law and its impact on HIV programmes for key populations

**DOI:** 10.3389/fpubh.2023.1272775

**Published:** 2023-10-18

**Authors:** Tendai Chikava, Rouzeh Eghtessadi, Innocent Chingombe, Grant Murewanhema, Alexander Cheza, Tafadzwa Dzinamarira, Helena Herrera, Godfrey N. Musuka

**Affiliations:** ^1^Independent Consultant, Independent Legal Consultancy Services, Harare, Zimbabwe; ^2^Technical Department, SAfAIDS Regional Office, Harare, Zimbabwe; ^3^Independent Consultant, Independent Public Health Consultancy, Harare, Zimbabwe; ^4^College of Medicine and Health Sciences, University of Zimbabwe, Harare, Zimbabwe; ^5^Discipline of Public Health, University of KwaZulu Natal, Durban, South Africa; ^6^School of Health Systems and Public Health, University of Pretoria, Pretoria, South Africa; ^7^School of Pharmacy and Biomedical Sciences, University of Portsmouth, Portsmouth, United Kingdom; ^8^Public Health Consultants, International Initiative for Impact Evaluation, Harare, Zimbabwe

**Keywords:** HIV, key population, Zimbabwe, law, female sex workers (FSW), men who have sex with men (MSM)

## Introduction

Zimbabwe is one of the four African countries to have achieved the UNAIDS 95-95-95 targets. However, significant programmatic gaps remain for selected key populations. Zimbabwe has several legal and policy constraints that hinder these key populations from seeking HIV prevention, treatment and care services. The criminalization of sex work and men who have sex with men are significant impediments to achieving 95-95-95 in the country. In this perspective, we explore how legal and policy constraints affect, in particular, men who have sex with men and female sex workers, who are most affected.

## Opinion

The latest UNAIDS report entitled “The path that ends AIDS: UNAIDS Global AIDS Update 2023” reports that Botswana, Eswatini, Rwanda, the United Republic of Tanzania and Zimbabwe have already achieved the 95–95–95 targets (1). Over the past decade, the successful HIV programmes in Zimbabwe have prioritized using evidence-based policies and approaches, resulting in increasing numbers of individuals accessing HIV prevention treatment and care, leading to major reductions in new HIV infections and HIV-related mortality.

However, key populations bear a disproportionately high burden of HIV in Zimbabwe. Men who have sex with Men (MSM) in Zimbabwe were found by a study to be at a higher risk of contracting HIV, with a 21% prevalence rate (2). In another study that was conducted in Zimbabwe, 50% of female sex workers (FSW) were found to be living with HIV, and < 50% of them had a suppressed viral load (3). This high burden is coupled with limited access to HIV services, mainly due to the criminalization of these activities.

As explained earlier, sex work and activities that support selling sex are criminalized. According to section 81 of the Zimbabwean Criminal Law (Codification and Reform) Act [Chapter 9:23], any person who publicly solicits another person for selling sex shall be guilty of soliciting and liable to a fine or imprisonment. Due to cultural imperatives and the patriarchal nature of society, male sex work is tolerated more than female sex work in the country. This perspective will, therefore, focus on female sex work. Due to stigma and legal repercussions, female sex workers and men who have sex with men do not always seek prevention and treatment services for HIV and sexually transmitted infections (STIs).

Evidence from the latest IBBS survey indicates that HIV prevalence among MSM is double that of the general population. This is in a context where same-sex relations are also prohibited by law, which includes the population of MSM, who, according to UNAIDS, are a key population for epidemic control. According to section 72 of the same Act, MSM is held to be committing a criminal offense. Owing to this criminalization, there has generally been very limited research on the dynamics of the HIV epidemic among this sub-group. The lack of information on the HIV epidemic among MSM, which is central to informing targeted response, has resulted from limited funding and interventions directed at HIV among this marginalized yet high-risk community.

Another example of a barrier to the generation of evidence-based to inform action relates to most new HIV infections in the country occurring in young women and girls. Parental consent is required to provide sexual and reproductive health and HIV testing services to individuals under 16 (4). This requirement is prohibitive for young female sex workers and MSM, who may need such services while not being keen to have their parents or guardians know about their sexual activities.

All Zimbabweans have a fundamental right to access primary healthcare. Nevertheless, as discussed earlier, the availability of these services is constrained by current legal and policy frameworks, which should be reconsidered. Parental and third-party consent restrictions for access to HIV prevention treatment and care services for FSW and MSM under sixteen remain a significant obstacle. Although obtaining parental approval may be a barrier to such services, the age of consent rules intend to safeguard youth (5). However, these constraints hamper much-needed access to HIV testing and services. Due to their fear of disclosure or abuse, parental consent restrictions may prevent young women from receiving vital sexual and reproductive health treatments.

Every human has the right to access health services despite their sexuality or whether they sell sex and this should be guarded particularly in what can be considered vulnerable key populations. The Constitution of Zimbabwe has a Bill of Rights which stipulates that everyone has a right to basic health care, including reproductive health care Criminalization and stigma are directly responsible for a reduced capability to effectively achieve and maintain epidemic control, and should be tackled as a priority. Zimbabwe needs to develop clear, laws that promote easier and timely access to HIV services. The law should not increase the vulnerability of certain individuals or groups, but should actively attempt to do the contrary and protect, particularly those individuals who need this the most.

## Author contributions

TC: Conceptualization, Writing—original draft. RE: Writing—review and editing. IC: Writing—review and editing. GMur: Writing—review and editing. AC: Writing—review and editing. TD: Writing—review and editing. HH: Writing—review and editing. GMus: Conceptualization, Writing—review and editing.
